# Direct gas-in-place measurements prove much higher production potential than expected for shale formations

**DOI:** 10.1038/s41598-021-90160-3

**Published:** 2021-05-24

**Authors:** Pedram Mahzari, Thomas M. Mitchell, Adrian P. Jones, Donald Westacott, Alberto Striolo

**Affiliations:** 1 Department of Earth Sciences, University College London, London, UK; 2 Halliburton, Houston, USA; 3 Department of Chemical Engineering, University College London, London, UK

**Keywords:** Geology, Geophysics, Petrology, Chemical engineering

## Abstract

Shale gas exploitation has been the game-changer in energy development of the past decade. However, the existing methods of estimating gas in place in deep formations suffer from large uncertainties. Here, we demonstrate, by using novel high-pressure experimental techniques, that the gas in place within deep shale gas reservoirs can be up to *five times* higher than that estimated by implementing industry standard approaches. We show that the error between our laboratory approach and the standard desorption test is higher for gases with heavier compositions, which are of strongest commercial interests. The proposed instrumentation is reliable for deep formations and, provides quick assessment of the potential for the gas in place, which could be useful for assessing hydrocarbon reservoirs, and the potential for geological carbon sequestration of a given formation.

## Introduction

Shale reservoirs and other tight rock gas resources have played a crucial role in the energy portfolio for the past two decades^1^. Knowing the realistic gas production potential of unconventional shale formations determines the ultimate recovery and gas rate of shale gas exploitation and its role on global energy security^2^. Uncertainty in the estimation of gas in place has been controversial in global shale gas developments^3–5^. For example, in the United Kingdom, the gas in place within the Bowland–Hodder shale formation was initially estimated as 1300 trillion cubic feet (TCF)^6,7^. However, subsequent studies resulted in much lower estimates for the volumes of gas in place, up to 6–7 times lower compared to former estimates^8^. In Poland, early-stage estimations indicated gas in place of 187 TCF^9^ whereas, recent figures suggest ~ 70 TCF^10^. Reliable estimates of the gas in place could reduce the financial and environmental risks associated with shale gas exploitation.

**Figure 1:**
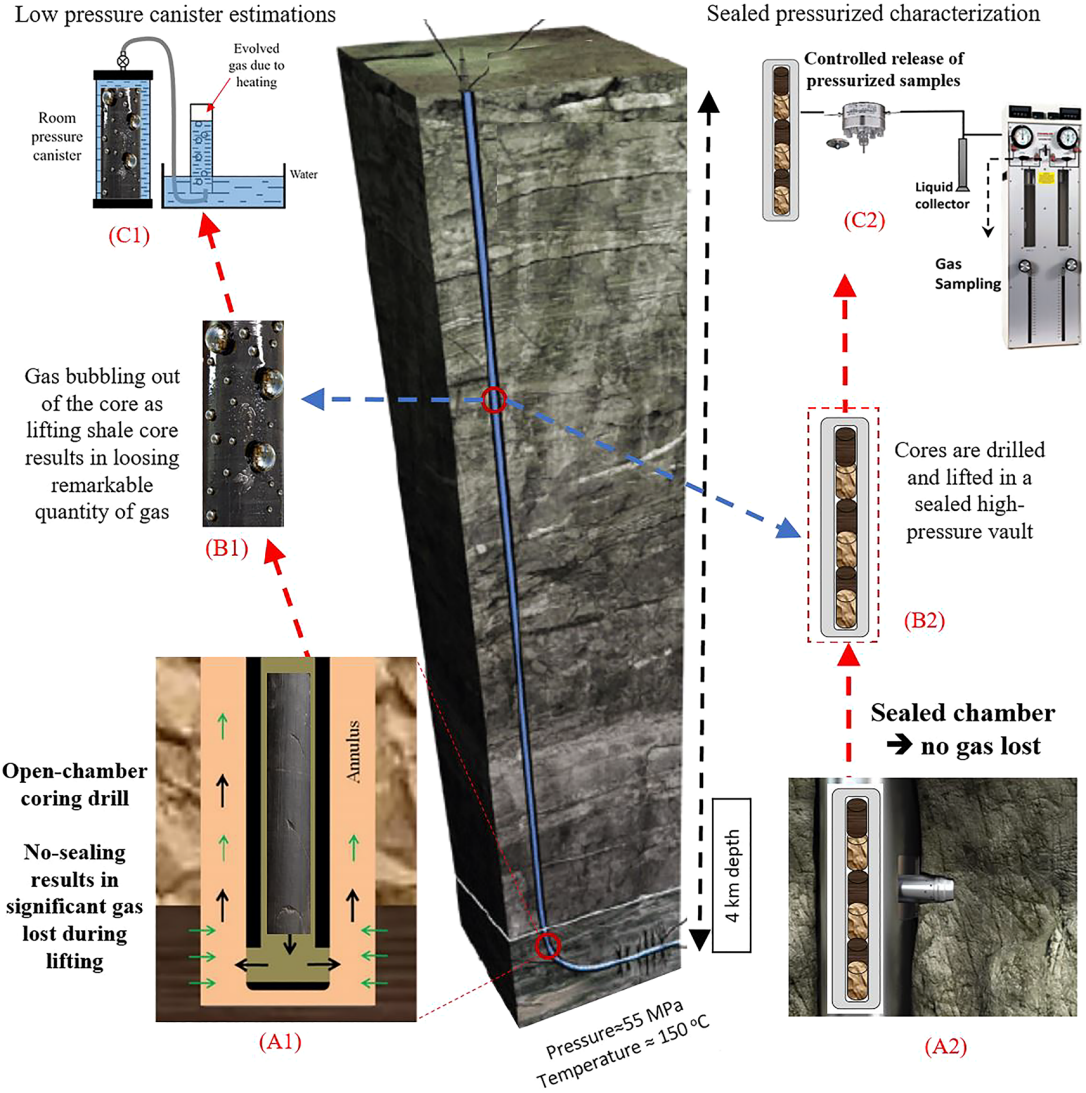
Estimation of gas content in shale reservoirs can be performed in two ways; (i) conventional low-pressure canister desorption (left hand side), rock samples release gas in transit before being analyzed at the surface (ii) side-wall rock cores are sealed at pressure before retrieval to the surface, in order to minimize the error of gas in place measurements.

In kerogen-rich shale rocks, hydrocarbon gases are contained within both pore volumes and adsorbed on the mineral surfaces, as illustrated in Fig. 1^11^. As the mineral surface area can accommodate significantly higher gas quantities compared to pore spaces (for instance, Bakken shale has ~ 2 $\frac{{m}^{2}}{gr}$ specific surface area and ~ 4 × 10^–9^ $\frac{{m}^{3}}{gr}$ of pore volume^12^), the amount of adsorbed gas plays a crucial role in accurate estimation of gas in place. Rock samples retrieved from deep geological reservoir formations are rightly considered as the best source of information to measure the gas content directly, which can then be used to calculate gas in place for a shale gas resource. It has long been recognized that, however, significant quantities of gas are lost during the core lifting stage, due mainly to transfer from high pressure and temperature to surface conditions, i.e.: decompression. Therefore, methodologies were adopted to calculate the lost gas during the core lifting. Standard methods for measuring the adsorbed gas volume rely on a laboratory-based technique known as canister desorption, which has been adopted from the coal bed methane industry^13,14^, where samples are taken from a well and then tested at ambient pressure. The USBM (United State Bureau of Mines) direct method is selected for desorption gas estimation as it is widely used in shale industry, which is suitable for high pressure conditions^14^. Other methods have also been proposed for gas desorption analysis of coalbed methane such as the Smith–William method, which may be more applicable for low pressure samples^15^. The Amoco method focusses on the decline period of gas desorption curves, which may not be directly linked to the core lifting process for deep shale gas formations^16^. During the core lifting process from deep shale gas formations, the gas lost would follow different trends compared to coalbed methane as shown in a simulation study by Wilson, et al.^17^. The fundamental difference between coal bed methane and shale gas resources is the significant contrast in the pressure regimes; coal bed methane formations are usually found in pressure ranges below 1000 psi (7 MPa) whereas shale gas reservoirs typically exist at 6–10 times higher pressure.

It is possible that some of the significant errors in estimation of gas in place may be attributed to inconsistency of ambient pressure evaluations of canister desorption tests to the much higher-pressure conditions of deep shale gas formations^14,18,19^. A key factor causing the discrepancies is therefore the lack of tailored laboratory methodology for accurately measuring the gas in place. Indeed, two laboratory methods are widely practiced: canister desorption tests and separate measurements of porosity and adsorption Langmuir capacities^7^. However, both approaches are known to produce notable degrees of error^8^, as reflected in the ~ sevenfold variability in range from 1300 to 185 TCF for UK shale gas resources. The complexity in estimation of the gas in place can be attributed to complex processes taking place during core lifting, which impact the adsorption characteristics of hydrocarbon gas components such as methane and ethane, where pressure and temperature conditions can have opposing impacts on adsorption behavior.

Having identified the discrepancies in estimation of gas in place, stakeholders have attempted to develop techniques which address the complexity of characterizing shale gas formations. A sidewall coring technology developed by Halliburton takes samples from target depths and delivers them to the surface in a sealed container maintaining the formation pressure from where it was sampled^20^. Hence, the fluids collected from the sealed chamber show a more representative estimation of the gas in place. The difference between high pressure sidewall core sampling and decompressed canister-type sampling at the surface is shown in Fig. 1. For low pressure canister estimations (as illustrated on the left-hand side of Fig. 1), the shale gas formation is cored using an open-chamber coring drill, the pressure of which is controlled by the head of the drilling fluid (A1 image). As the core sample is lifted towards the surface, the pressure drop leads to gas release, loosing significant amounts of hydrocarbon gas (B1 image). Once the shale sample is retrieved at the surface, routine canister desorption tests are performed to estimate the lost gas (C1 image). By contrast, the sealed pressurized characterization approach (right-hand side of Fig. 1) can acquire the shale core from multiple depths of the formation using side-wall coring bits (A2 image). The bit is designed to minimize the impacts of the well drilling and drilling mud since the coring bit can acquire the core sample in-situ once the well primary drilling is completed. The shale gas side-wall cores are drilled under reservoir pressure from selected locations, and stored in a sealed vessel, which maintains the pressure and fluids intact (B2 image). As the sealed vessel is lifted to the surface and transferred to laboratory, the fluids can be collected under controlled release of pressure (C2 image). In this work, the results of the laboratory replication of the two approaches are investigated and compared with field observation of the gas in place estimations.

Here, we describe a new experimental system, developed to simulate the deep subsurface sidewall core retrieval process^21,22^ and to accurately measure gas volumes extracted from natural high-pressure shale samples. Our experiments recreate the subsurface conditions for pressure (P) and temperature (T) of deep commercial shale formations; subsequently, the core lifting process is applied to the cores by controlled decompression. Initially, preserved waxed core samples from the well-studied Haynesville–Bossier formations were used to be pressurized with multi-component reservoir gas. Then, the pressure and temperature conditions of core lifting were mimicked to measure the lost gas during core retrieval^17^. The newly derived total gas in place was compared with the results of routine methods such as canister desorption tests and reveals significantly higher quantities of gas in place in the pressurized shale samples. Subsequently, the results of the laboratory experiments are compared with pressurized sidewall coring, which were carried out in a producing shale gas formation. The findings from the laboratory experiments are in good agreement with results of pressurized sidewall coring in the field as performed on deep shale gas formations.

## Methodology

**Figure 2:**
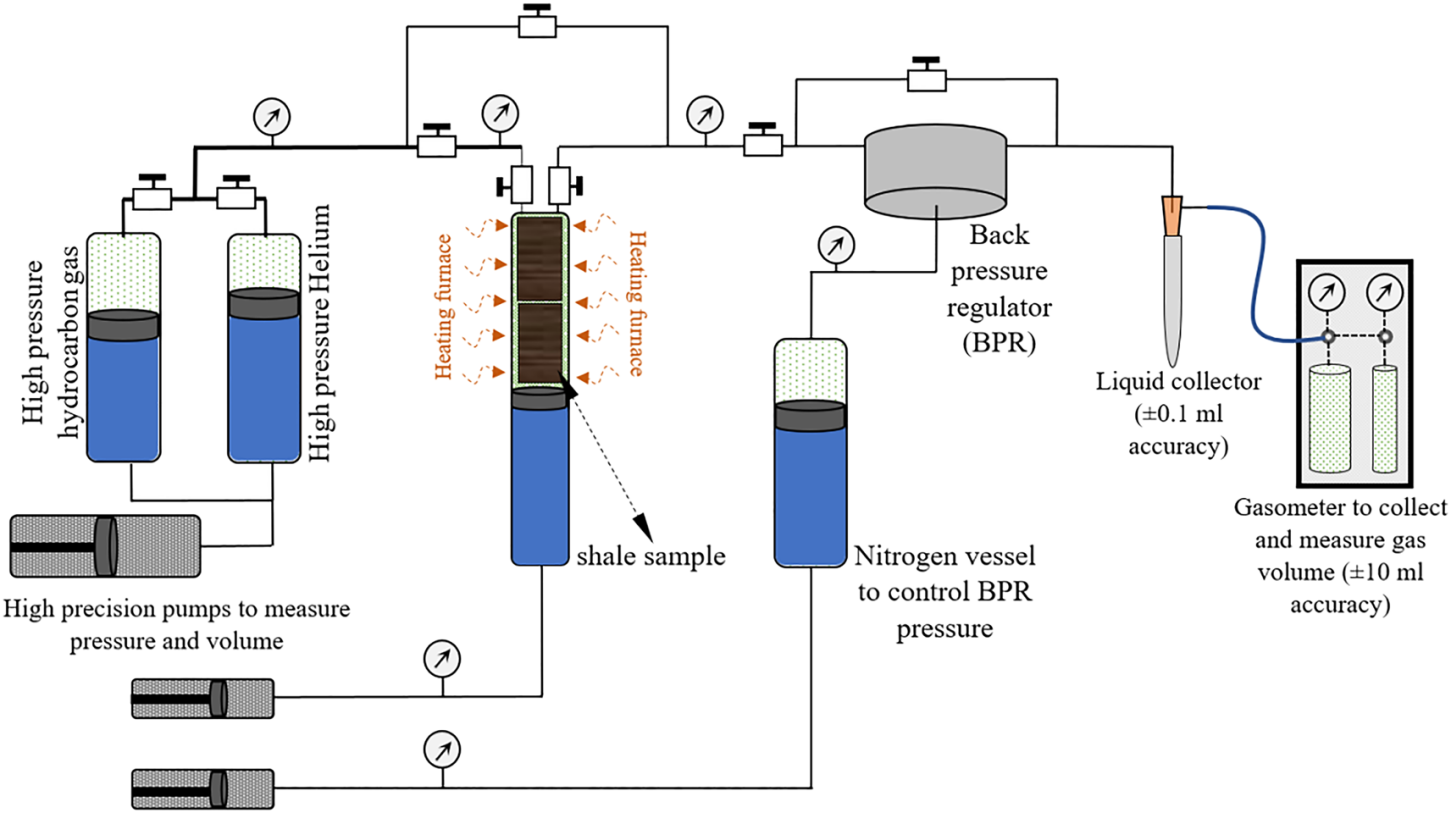
Schematic of laboratory apparatus for direct measurement of gas in place for shale samples. See text for details.

**Figure 3:**
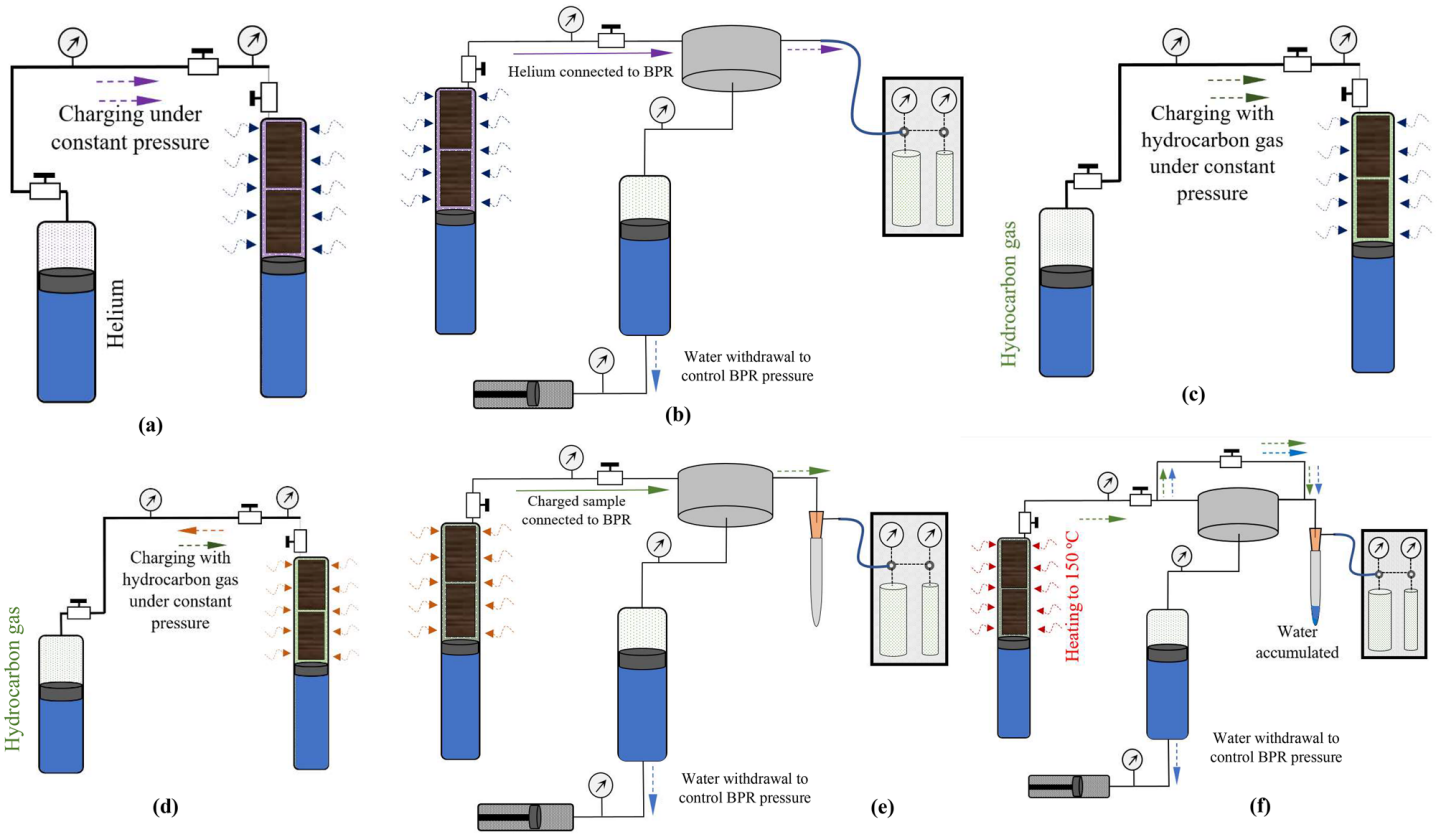
Experimental procedure of controlled decompression experiments for gas in place measurements: (**a**) helium calibration of pore- and dead-volumes under low-T; (**b**) measuring charged helium at low decompression rate; (**c**) charging with hydrocarbon at low-T; (**d**) applying reservoir temperature; (**e**) producing the hydrocarbon gas under core-lifting conditions; (**f**) heating up the core to 150 °C to measure gas and water. This last stage replicates the conventional canister desorption.

The rock core samples are placed in a high-pressure vessel, which is connected to a two-vessel configuration for helium and hydrocarbon gas tanks, which can be used to accurately charge, or re-charge gas into the sample at the desired pressure. The charging cylinder is equipped with a heating furnace to apply reservoir temperature to the core samples (Fig. 2). The apparatus is equipped with a nitrogen cell to accurately control the decompression processes. The effluent is passed through a separator or liquid-collector to measure the condensed liquid constituents of the evolved gas. Once the sample has been equilibrated with gases, it can be decompressed at controlled rates. The decompressed gas volumes are collected in a gasometer with accuracy of ± 10 ml at room temperature and pressure conditions. The procedure first requires physical analysis of the sample in the charging cylinder to measure the dead and pore volumes, using helium as a non-adsorbing gas at low 30 °C temperature (Fig. 3a). Saturation with helium is identified from stable pressure transducers, then helium is passed into a gasometer to measure its volume (Fig. 3b). During this stage, a small but negligible degree of hydrocarbon gas desorption could occur. This slightly higher temperature can desorb some gas content as it heats the core by 10 °C. No noticeable amount of gas could be collected by this heating during our experiments. The charging and decompressing stages for helium were performed under very slow pressure rise and decline rates (10 $\frac{psi}{min}$) to minimize rock dilation and compression effects.

Then, hydrocarbon gases matching the shale gas were used to charge the shale cores up to the lithostatic pressure of the target underground shale reservoir formation (Fig. 3c). For the experiments reported in this study, the shales were pressurized to 6000 psi, which is the average pressure of the shale formation used here. Once the sample is charged at the reservoir pressure, the temperature of the main vessel is increased (Fig. 3d). This step can cause exchange of the gas components between the hydrocarbon vessel and the main vessel due to gas expansion and varying relative adsorbing capacity of hydrocarbon gases at different temperatures. At higher temperatures of up to 150 °C for the test performed in this study, hydrocarbon components can be desorbed, although it is known that the desorption hysteresis tends to be more pronounced for heavier gas components^23^. Because of possible hysteresis, and also because of the typical low permeability of shale rock samples, the experimental step necessary for establishing the reservoir pressure and temperature can take up to 3 weeks. The pressurized shale rock was then connected through the back-pressure regulator to impose pressure and temperature conditions of core lifting (Fig. 3e). Therefore, the lost gas during the core lifting process can be directly measured and compared with other proposed methods such as canister desorption tests. After performing the core lifting scenario, the canister desorption test was carried out by heating the shale cores to higher temperatures under ambient pressure (Fig. 3f).

## Results

**Figure 4:**
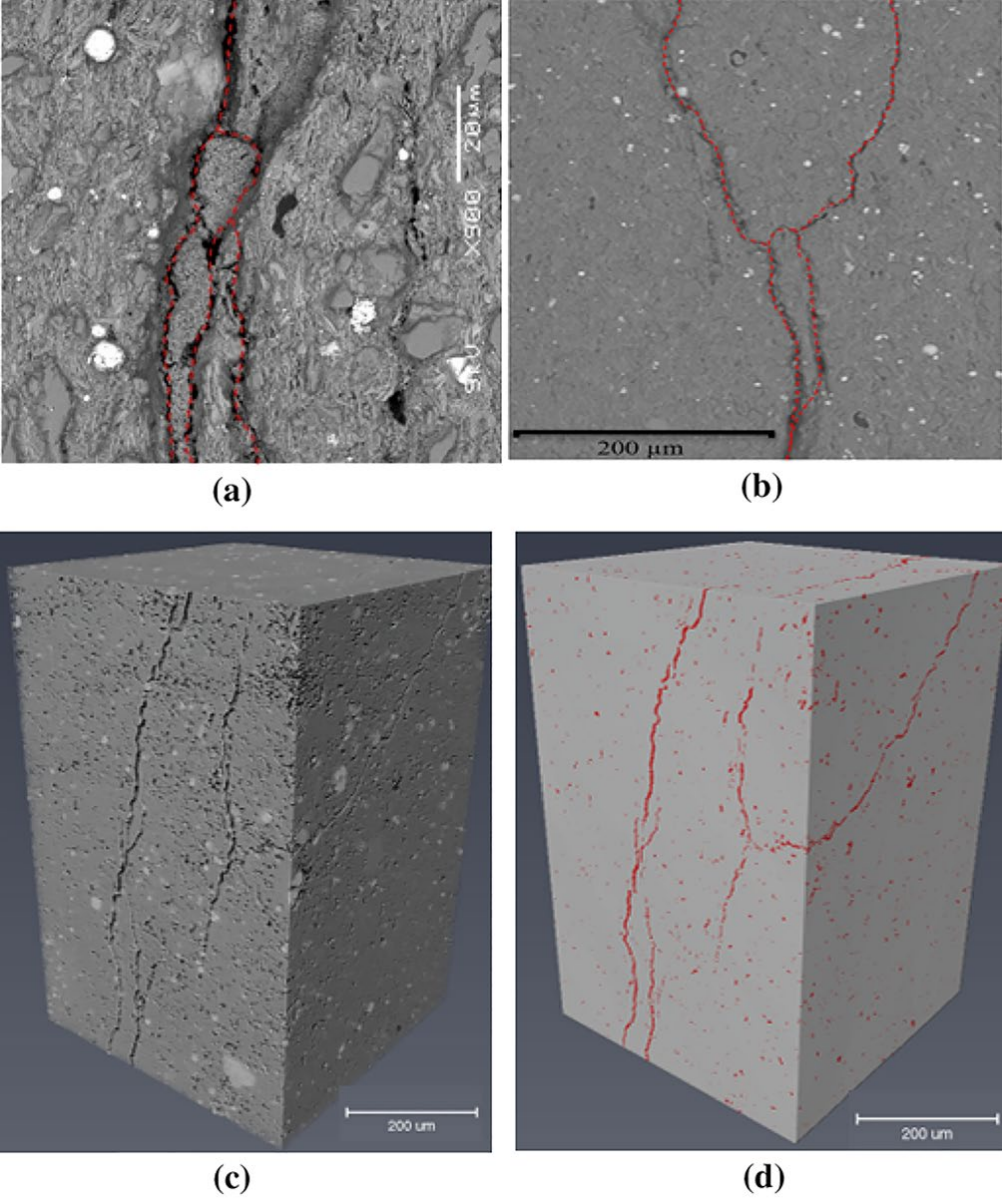
Observations of the shale core used for experiments. (**a**) SEM image of a rock chip. (**b**) SEM image of a microfracture located on the top edge of the core, (**c**) 3D perspective micro-CT X-ray image of the core cut from top. (**d**) Segmented volume micro-CT image; the void porosity and micro-fractures as highlighted in red.

Four shale samples, cored using a reference shale core block of the Haynesville–Bossier formation from a depth of 11,750 ft, were used for the experiments; two for canister desorption tests and two for high pressure charging^21,22^. Two similar cores for canister testing were used in order to confirm repeatability of the results. Out of the other two cores, one is used for charging with methane and one with multicomponent gas, to assess the effect of gas chemical composition on the estimation technique. Details of average core properties can be found in Table 1. A chipping from shale whole core was examined for detailed pore-scale imaging. SEM images were combined with micro-CT X-ray imaging to characterize pore-scale characteristics of the shale sample (Fig. 4). Figure 4a,b depicts SEM images of a polished piece of the shale sample. The white spots on the SEM images indicate pyrite while the grey regions represent quartz and carbonates. Figure 4b also shows that the micro-fractures are connected, creating highly laminated rock samples. A chip was used for micro-CT X-ray imaging using a micron-scale Zeiss Xradia 520 Versa instrument 57. Figure 4c shows a micro-CT image of this core chip while Fig. 4d depicts the same image with the void and fractures highlighted in red; the latter was obtained from a segmented volume image with voxel size of 1000 × 1000 × 1500^24^. From analyzing the micro-CT images, we estimate that the sample has an average micro-fracture porosity of 2.5%. To quantify the gas composition, we conducted gas chromatography tests for the produced gas from the shale samples, the multicomponent gas was composed of methane, ethane, propane, carbon dioxide, and nitrogen, with compositions as listed in Table 2. The multi-component gas is expected to produce results closer to the natural system, since it can capture the complex relative adsorption and desorption characteristics of mixed organic gas components in natural gas^24^.

**Table 1:** Basic properties of shale gas cores used in the laboratory measurements^21,24^.

Core ID	Mineralogy (weight%)	Porosity (fraction)	TOC (wt%)	Matrix permeability (mD)
Bossier–Haynesville core samples	Clay = 40 ± 8 Carbonates = 10 ± 7	0.07–0.08	3.2 ± 0.4	3.56 × 10^–4^ (samples have micro-fractures)

**Table 2:** Composition of hydrocarbon gas used for charging the shale samples.

Component	Composition (weight percent)
Nitrogen	1.1
Carbon Dioxide	2.3
Methane	91.8
Ethane	4.4
Propane	0.4

The estimated critical point of the multicomponent gas is 1070 psi and 31 °C using Peng Robinson equation of state. Hence, the hydrocarbon phase is supercritical at reservoir conditions and vapor at room conditions.

**Figure 5:**
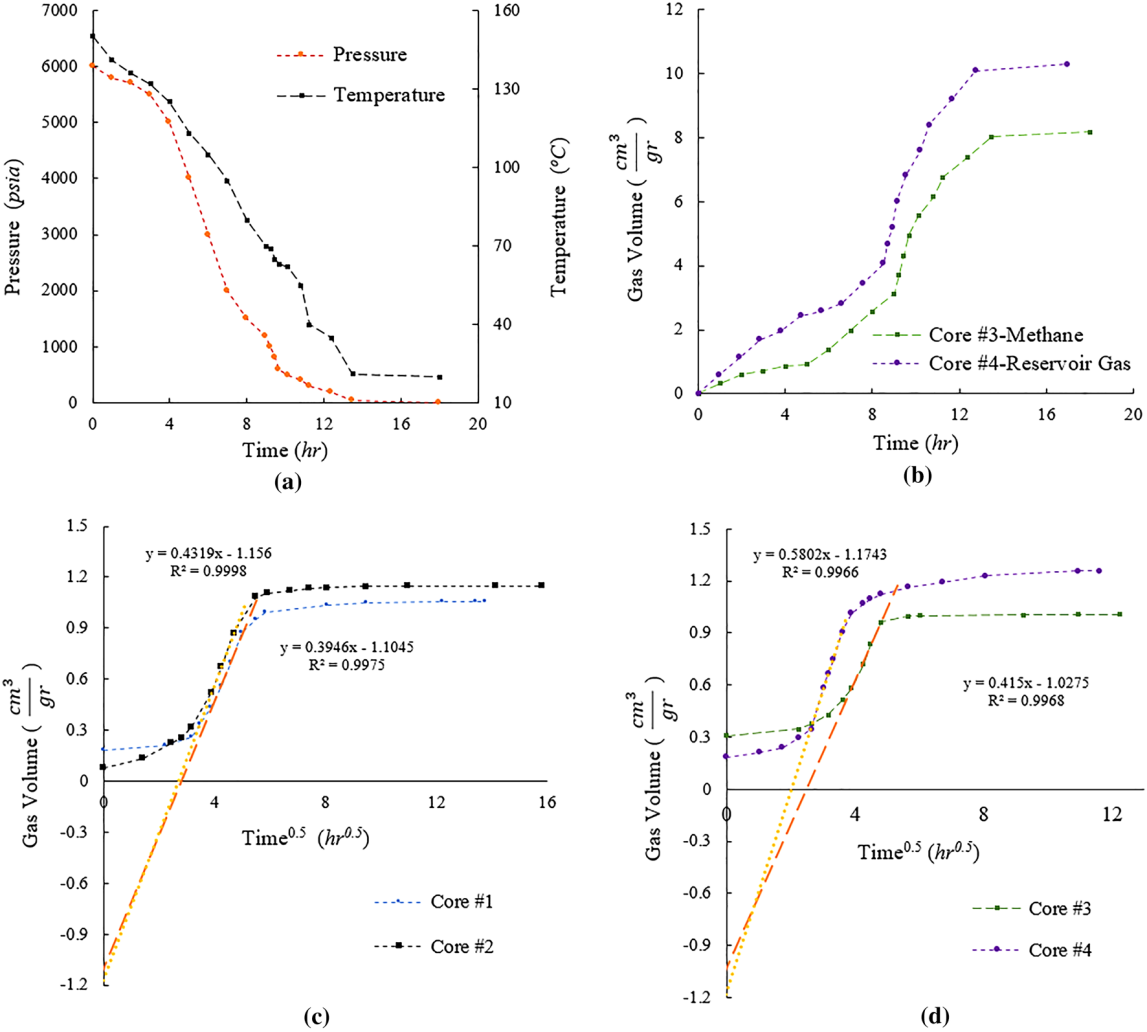
Results of laboratory experiments performed on 4 shale core samples (Cores#1–4). (**c**) The temporal profiles of gas volume from canister tests performed on Core #1 and #2. (**a**) the pressure and temperature drops imposed on the high-pressure cores, i.e. Core #3 and #4, which is relevant to core lifting process. (**b**) The gas volumes collected during decompressing period. (**d**) the canister tests were performed on the charged samples and the temporal gas volume profiles are depicted. For canister test the total gas recovered is in range of 450–500 ml which is 2% of measurement uncertainty. For the pressurized samples, the total gas recovered is 2000–2500 ml, which leads to measurement uncertainty of 0.5%.

The degassing experiments performed on four shale cores were monitored continuously as a function of time to ensure that pressure and temperature changes closely represent the core lifting process in the field. The measured gas volume released during the simulated core lifting closely follows the decompression curves (Fig. 5a,b). All estimated gas volumes are normalized to shale core mass to ensure comparability of the results. Based on the standard USBM method^14^, the linear part of the canister tests results can be extrapolated to estimate the lost gas volume. Lost gas refers to the gas which escaped from the core during the core lifting process.

The gas evolved by heating to 150 °C represents the canister desorption test, where similar results indicate reproducibility of the experiments for the reference shale core (Fig. 5c). Canister desorption tests performed on the pre-pressurized cores, but after the decompressing stage, yield different amounts of lost gas (i.e. intercept of the extrapolated line: Fig. 5d) for samples with different gas chemical compositions. A summary of the gas in place estimations as measured by all techniques just discussed is presented in Table 3. It should be pointed out that heating the preserved shale gas samples could lead to accumulation of water in the liquid collector, which represented an average water saturation of 18% in the shale rock samples.

**Table 3:** Gas in place measurements by high pressure charging (decompressed) and standard canister methods.

Core ID	Gas used	Desorbed gas volume (canister test)	Decompressed gas volume	Total gas by canister	Total gas by decompressing	Error
		ml/gr	ml/gr	ml/gr	ml/gr	
Core 1		1.05	–	2.15	–	–
Core 2		1.15	–	2.30	–	–
Core 3	Methane	1.00	8.18	2.02	9.18	450%
Core 4	Multicomponent gas	1.25	10.29	2.42	11.54	480%

The gas volumes are normalized by weight of shale rock samples. The error presented in the last column is the difference between decompressed and canister volumes divided by the decompressed gas volume.

**Figure 6:**
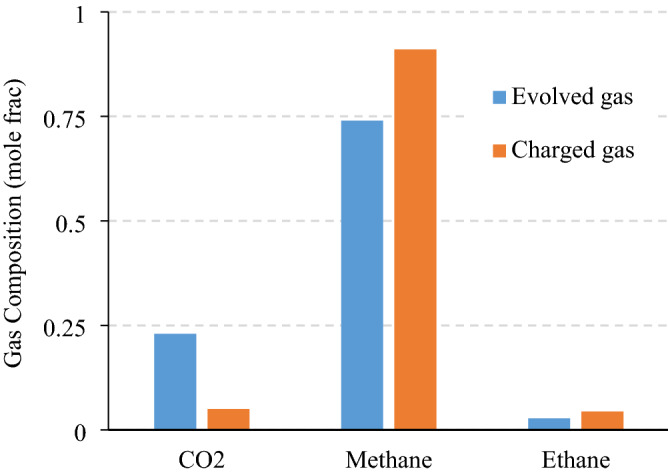
Composition of charged and evolved gases used and collected, respectively. The evolved or desorbed gas contained more CO_2_ compared to the charged gas.

The laboratory-controlled pressure and temperature profiles represent 18-h of core lifting (Fig. 5a) during which time the gas within the shale cores would have escaped or been lost in transit for natural samples in the field. This is likely to be the main source of error for gas in place estimations when the industry-standard approaches are implemented in the field. Although the temperature and pressure profiles could resemble a linear trend for the first 13 h, the profiles of gas escape or gas lost indicate a non-linear behavior (Fig. 5b). The first 8 h of decompressing exhibits a slower gas escape while the pressure drops from 6000 psia to 1500 psi, increasing notably once pressure and temperature drops below 1500 psi and 70 °C. Such non-linear behavior took place consistently for both shale cores. This finding is in agreement with adsorption isotherms measured for different hydrocarbon components where gas adsorption plateaus at pressures above 2000 psi^11^. Moreover, this observation can be used to infer the depth of core lifting associated with the highest portion of the gas lost, and which corresponds to relatively shallow depths. For the charging and decompressing experiments, methane and multi-components gases were used and the results indicate ~ 20% higher gas lost for multi-component gas, which is more representative of the realistic shale gas resources. To be able to compare canister tests with our new pressurized methodology, Fig. 5c shows a gas evolution of approximately 1 $\frac{{cm}^{3}}{gr}$ whereas the gas lost of pressurized samples was around 10 $\frac{{cm}^{3}}{gr}$. This highlights the fact that the samples lifted to surface are expected to contain substantially lower gas volumes compared to the subsurface shale reservoir formation. The lost gas estimated from canister experiments by the intercept of the extrapolation of the linear part of the profiles (Fig. 5c) is approximately 1 $\frac{{cm}^{3}}{gr}$, which is only 10% of the true lost gas measured in decompressing test (Fig. 5b). Once the charged cores (i.e. Core #3 and Core#4) experienced the core lifting conditions, the cores were heated to mimic the canister tests on retrieved cores (Fig. 5d). The gas evolution profiles recorded for Core #3 and #4 are similar to that obtained for Core #1 and #2, which indicates consistency in the canister desorption measurements between the pristine cores and the charged cores after performing the mimicked core lifting processes. If the results of the decompression experiments were compared against the canister tests, it is conceivable to encounter an 480% error of gas in place estimation, as listed in Table 3. The gas composition for the reservoir gas and the evolved gas after canister desorption can be significantly different due to desorption capacity of gas components. For the experiments performed on Core 4, the gas collected during canister desorption heating was analyzed by gas chromatography. Figure 6 compares the gas composition used for charging the shale sample with the gas composition of the desorbed gas at the last stage of heating at room pressure Evidently, the desorbed gas by canister heating did contain more CO_2_ and less methane compared to charged gas. The higher content of CO_2_ in the desorbed gas can be attributed to higher adsorption propensity of CO_2_ compared to methane^25^. For heavier components such as ethane and propane, the difference in the two gases was not as high as that of CO_2_ and methane. This difference in composition can also indicate that, the low-pressure gas release by heating can target a gas with different characteristics, which can introduce notable errors in gas in place estimation. On the other hand, this notable remaining CO_2_ within the shale sample after depressurization due to excess adsorption of CO_2_ can indicate the considerable capacity of the shale rocks to house CO_2_ during various applications of CO_2_ injection such as EOR huff-n-puff and CO_2_ storage^24,26,27^.

## Discussion and conclusions

Our results show that the gas volume stored in the shale core samples can be 4.5 to 5 times higher for pressurized samples, compared to that of industry-standard canister estimations. The difference is because the lost gas in transit is not taken into account in the current methodologies. The common neglect of this gas contribution, lost in transit during decompression, can likely be attributed to the inconsistency of the gas-in-place estimates reported in the literature for high pressure shale formations. We note that the standard canister test was adapted from coal-bed methane to shale resources, while there is a fundamental difference between pressure regimes of coal-bed methane and deep shale formations. This remarkable difference in estimation of gas in place could significantly change the prospect of developing shale formations, in terms of quantifying total volumes in place. It may also explain why the drawdown of gas from some shale reservoirs, carries on for much longer durations. While the measurements presented in this work have focused on core scale gas in place, the results of laboratory measurements can be upscaled to the size of the shale formation to obtain the total cubic feet of gas in place in the shale play.

Our results suggest that the gas composition could notably affect the second-order calculations of the gas in place since, as we have shown, multicomponent gas, which is more similar to natural gas, has a higher degree of adsorption compared to pure methane^11^. Therefore, as the gas composition becomes enriched in heavier components such as ethane and propane, the relative proportion of lost gas unattributed by the canister estimation, would tend to be higher. One advantage of this laboratory protocol is the capability of studying the partially depleted formations; for those cases, the high pressure charged samples can be brought to current shale gas pressure under controlled drawdown and then, the remaining gas in place can be directly measured via following the same procedure explained for mimicked core lifting processes.

To compare the results of the laboratory experiments with field observations, we show the outcome of deploying multiple pressurized side-wall cores retrieved from various depths in a shale formation using a commercial tool described in Fig. 1, which was designed to prevent gas loss in transit (Table 4). The side-wall coring was extensively performed on the Marcellus gas shale formation, which followed the procedure explained in Fig. 1 (right-hand side approach). In Table 4, the results of the high pressure sealed coring for shale samples with similar TOC are provided. The high-pressure side-wall cores yield 4–6 times higher gas volume compared to that estimated by canister tests. The discrepancies can be attributed to pressure and temperature conditions and core properties such as total organic content. The overlap in results listed in Tables 3 and 4 are strikingly similar. Therefore, many commercial shale gas formations could have significantly higher quantities of gas in place, missed by the reliance on standard canister testing, which provides an underestimate. The agreement between the laboratory measurements and field based high-pressure side-wall coring, demonstrate the robustness of experimental methodology.

**Table 4:** Results of gas in place measurements by pressurized side-wall cores and standard canister methods.

Reservoir ID	Average depth	Decompressed gas volume using pressure release	Desorption volume using canister heating	Error of canister test (%)
	feet	ml/gr	ml/gr	
Shale 1	6600	3.71	0.61	604.15
Shale 2	8050	0.93	0.16	588.15
Shale 3	8400	5.62	1.31	429.48
Shale 4	14,700	2.08	0.37	555.53

The side-wall coring was deployed in Marcellus formation.

Our results suggest the gas in place for shale formations could be consistently higher than estimated using canister tests alone. In the chosen field example, current estimates of gas in place for the Bowland Shale, UK lie between 1300 to 186 trillion cubic feet, which is a highly uncertain range. Indeed, the discrepancy in the gas in place likely stems primarily from the uncertainty in the core scale measurements^6,7^. Using crushed rock samples to measure adsorption isotherms can also be misleading, as the crushed rock could exhibit a biased surface rock mineralogy and surface morphology^23^.

In summary, our results of direct laboratory experiments and high-pressure sidewall coring support higher shale gas potentials compared to that estimated by conventional canister tests. Shale gas-in-place estimation has been a challenge and, we have proposed a reliable experimental approach that agrees with new technologies deployed in the fields. NMR and other methods are well suited for characterizing various types of porosities in shale samples^28^. Indeed, although our proposed technique does not measure porosity, it is able to estimate accurately, quickly and cheaply the gas storage potential of challenging rock samples such as shales. This is fundamentally due to gas lost by decompression during retrieval to the surface. Like a famous quote, “if the horses have left the barn, it’s difficult to coax them back to the stable”, the lost gas during shale core lifting is virtually impossible to estimate from a standard USBM canister method. The error of current estimation methods can reach to up to 450–480%. Add to subsurface geological extension of a shale formation, the error may be even greater increased when upscaled. More importantly, evaluation of a shale play depends primarily on the core scale estimation of gas in place and representative estimation of gas in place is required prior to development plans. Our results highlight the fact that, tailored methodologies for analyzing the high-pressure samples are essential for a sound evaluation of a shale gas reservoir. What we propose for reliable laboratory characterization of gas in place is to basically impose the high pressure reservoir conditions on pristine shale cores using reservoir gases and then, mimic the core lifting process. Our methodology can be an alternative for robust direct gas in place measurements.
